# Supplementary material for: The causal relationship between the human gut microbiota and pyogenic arthritis: a Mendelian randomization study
Source: Front Cell Infect Microbiol. 2024 Nov 26;14:1452480. doi: 10.3389/fcimb.2024.1452480 (PMC11629706; doi:10.3389/fcimb.2024.1452480)
Supplement: Supplementary file 1 [file DataSheet1.zip › mendelian test/ebi-a-GCST90027583.csv_forest.pdf]

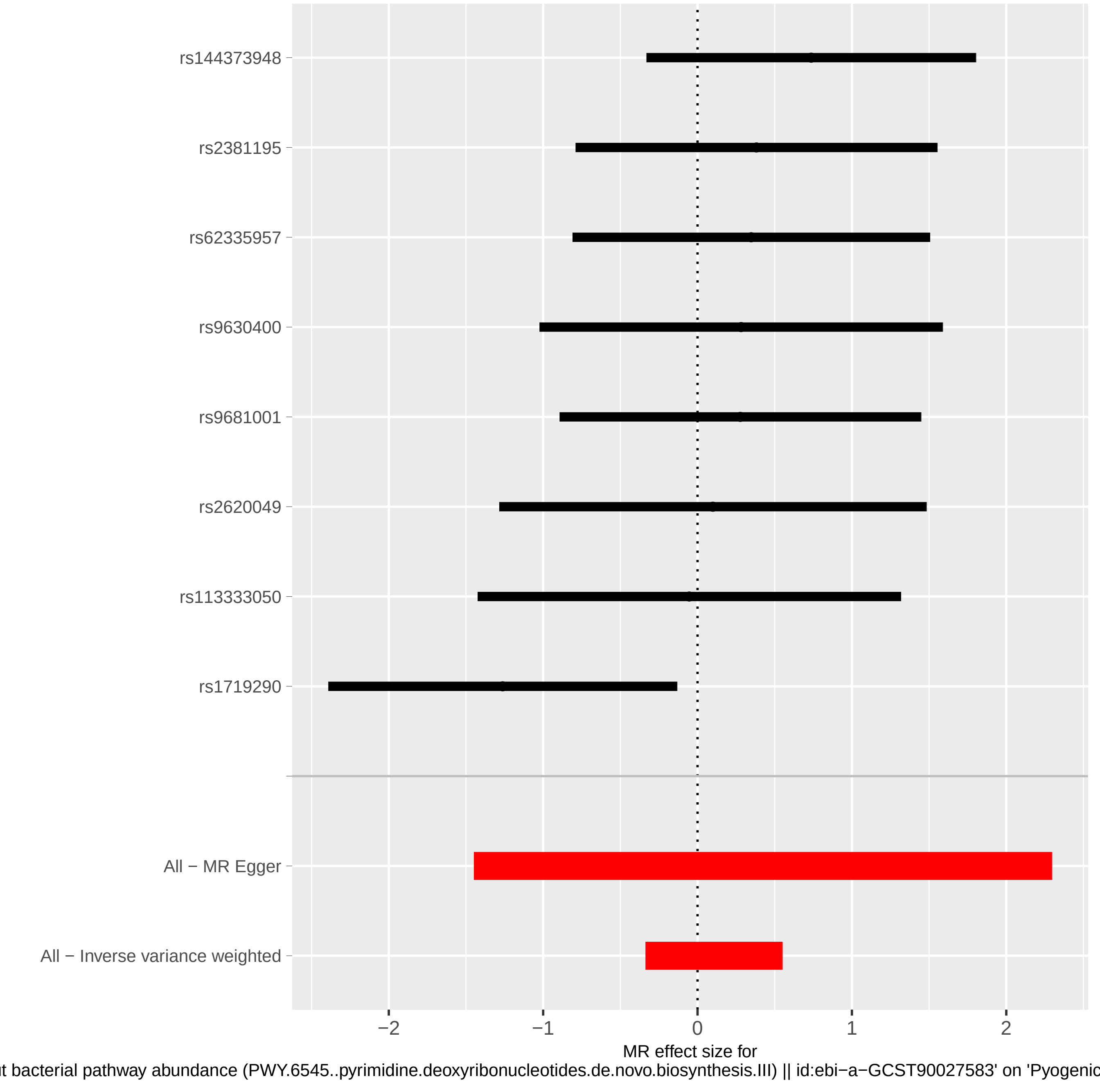
rs144373948
rs2381195
rs62335957
rs9630400
rs9681001
rs2620049
rs113333050
rs1719290
All − MR Egger
All − Inverse variance weighted
−2
−1
0
1
2
MR effect size for
t bacterial pathway abundance (PWY.6545..pyrimidine.deoxyribonucleotides.de.novo.biosynthesis.III) || id:ebi−a−GCST90027583' on 'Pyogenic
